# Parenteral nutritional support in surgical patients: expert consensus statements regarding intravenous lipid emulsions containing omega-3 fatty acids

**DOI:** 10.3389/fnut.2025.1546089

**Published:** 2025-02-17

**Authors:** Robert G. Martindale, David C. Evans, Dan Waitzberg, Malissa Warren, Manpreet S. Mundi, Stanislaw Klek, Paul E. Wischmeyer, Martin D. Rosenthal

**Affiliations:** ^1^Department of Surgery, Oregon Health and Science University, Portland, OR, United States; ^2^Nutrition Support Service, OhioHealth, Columbus, OH, United States; ^3^Department of Gastroenterology (LIM-35), School of Medicine, University of São Paulo, São Paulo, Brazil; ^4^Nutrition and Food Service Portland, VA Health Care System, Portland, OR, United States; ^5^Division of Endocrinology, Diabetes, Metabolism and Nutrition, Mayo Clinic, Rochester, MN, United States; ^6^Surgical Oncology Clinic, The Maria Sklodowska-Curie National Cancer Institute, Krakow, Poland; ^7^Department of Anesthesiology and Surgery, Duke University Hospital, Durham, NC, United States; ^8^Department of Surgery, Division of Trauma and Acute Care Surgery, University of Florida College of Medicine, Gainesville, FL, United States

**Keywords:** consensus, fish oil, guidelines, lipids, omega-3 fatty acids, parenteral nutrition, surgery

## Abstract

**Objectives:**

The International Lipids in Parenteral Nutrition (PN) Summit was convened to offer practical guidance and expert consensus opinion regarding the use of intravenous lipid emulsions (ILEs) in various clinical settings. Herein, we briefly review aspects from this summit that are of particular importance for surgical/hospitalized patients.

**Methods:**

Summit participants identified and discussed new evidence, data, and analyses, that potentially influence the benefits and risks of ILEs in PN or their use in clinical practice. The summit meeting consisted of expert presentations that assessed recent clinical data and best practice, followed by periodic panel discussions to formulate consensus statements. Consensus statements were voted on, anonymously, by the meeting attendees.

**Results:**

This review briefly summarizes the rationale for considering ILE choice as a central component of any PN strategy for surgical/hospitalized patients. Thereafter, special patient populations are considered, such as surgery-related intestinal failure, major trauma, and those with chronic critical illnesses. Expert consensus statements are also provided to help bridge the gaps between evidence and clinical practice, hence complementing formal PN societal guideline recommendations.

**Conclusion:**

The choice of ILE in PN, particularly those containing fish oil, can play a vital role in improving outcomes for surgical patients.

## Introduction

The trauma of major and even minor surgery, induces physiological changes including the activation of inflammatory and catabolic pathways, which can affect patient recovery (1). However, optimal nutritional status helps the body to recover in a faster and more efficient manner from these stressors, whereas malnutrition can lead to worse surgical outcomes and to a higher likelihood of postoperative complications (e.g., infections, delayed wound healing, pressure lesions, respiratory failure, increased length of hospitalization, and higher mortality rates) (1). As such, the importance of nutrition for surgical patients has long been recognized, and includes the need to assess patients’ nutritional status before and after major surgery, the requirement to provide nutritional support for those who are malnourished or who are at nutritional risk, and evolve nutritional strategies for enhanced recovery after surgery (2).

In practice, however, up to 2 out of every 3 patients presenting for major surgery are malnourished pre-operatively, and there is still often an imbalance between energy and nutrient requirements and patients’ actual intake, such that surgical patients with malnutrition continue to be underdiagnosed and undertreated (3). Parenteral nutrition (PN) is generally considered to be a way of supplying energy and nutrients in situations where enteral or oral nutrition is not possible or is insufficient (2). Nonetheless, another important factor to consider as part of any PN strategy is the choice of intravenous lipid emulsion (ILE), because this can have physiological effects and influence clinical outcomes. For example, fish oil is rich in omega-3 polyunsaturated fatty acids (PUFAs), which have a role in the active resolution of inflammation without impairing host defense mechanisms, and may facilitate post-surgical recovery and help to preserve muscle mass (4). Thus, ILEs containing fish oil have been associated with improved clinical outcomes, such as reduced infection rates and shorter hospital and ICU stays (5–7). In contrast, pure soybean-oil ILEs, which have been in use since the early 1960s, have a relatively high omega-6 PUFA content. The metabolic derivatives of omega-6 PUFAs (e.g., linoleic acid) can adversely affect the immuno-inflammatory response to a traumatic insult, shifting the surgical patient towards toward a hyperinflammatory yet immunosuppressed state, that further drives catabolism (8, 9). Given these issues, this review examines the reasons for such nutritional imbalances and potential solutions, particularly from a perspective of PN and ILE choice, discussing potential controversies and offering practical guidance for surgeons.

In response to these clinical complexities, the International Lipids in Parenteral Nutrition Summit was held on November 3 and 4, 2022, in New Orleans, USA, bringing together experts with clinical and scientific experience of PN to discuss biological and clinical aspects of lipids used in PN (10). Consensus statements were produced to provide practical guidance regarding the use of intravenous lipid emulsions (ILEs) in PN that complement societal nutrition guidelines encompassing surgical patients (2, 11, 12). Table 1 shows consensus statements from the international Lipids in Parenteral Nutrition Summit relevant to the surgical setting. Furthermore, two case reports are presented in this review article to help underscore key learning points from the consensus statements and the summit. For further details in methods used to formulate the consensus statements, please see prior publications also based on this summit concerned with scientific aspects of the meeting (4) and translating guidelines into clinical practice (10). The authors of this review were responsible for the information presented here.

**Table 1:** Consensus statements for intravenous lipid emulsion (ILE) use in adults receiving parenteral nutrition (PN) relevant to surgical patients.^a^

Consensus statement	Voting
Medical and surgical ICU patients	
6. In adult medical and surgical ICU patients requiring parenteral nutrition, ILEs are an integral part of parenteral nutrition.	Agree: 18 (100%) Do not agree: 0 Do not wish to answer: 0
7. The use of ILEs containing fish oil should be considered during the first week of ICU admission in adult patients requiring parenteral nutrition, including surgical and critically ill patients, based on biological plausibility and associated clinical benefits.	Agree: 18 (100%) Do not agree: 0 Do not wish to answer: 0
8. There is accumulating scientific evidence from clinical trials, systematic reviews, and meta-analyses to demonstrate that ILEs containing fish oil have clinically meaningful advantages over ILEs without fish oil when used in adult patients requiring parenteral nutrition including surgical and critically ill patients with a favorable risk–benefit ratio. Furthermore, the use of ILEs containing fish oil as part of parenteral nutrition has shown to be cost-effective in these populations.	Agree: 17 (94.4%) Do not agree: 0 Do not wish to answer: 1 (5.6%)
9. In adult medical and surgical ICU patients, the total lipid dose is in general up to 1.5 g lipids/kg/day (including non-nutritive lipid sources such as propofol). A minimum ILE dose should be given to prevent EFA deficiency.	Agree: 17 (94.4%) Do not agree: 1 (5.6%) Do not wish to answer: 0
10. Based on currently available clinical data, we recommend 0.1–0.2 g fish oil/kg/day, provided by ILEs containing fish oil, for adult medical and surgical ICU patients requiring parenteral nutrition.	Agree: 17 (94.4%) Do not agree: 0 Do not wish to answer: 1 (5.6%)
11. The use of mixed lipid emulsions containing soybean oil, olive oil, MCTs and/or fish oil at the recommended dose has not been shown to lead to EFAD in clinical practice. A 100% fish oil ILE has also not been shown to lead to EFAD in clinical practice.	Agree: 18 (100%) Do not agree: 0 Do not wish to answer: 0
12. The concentrations of triglycerides in serum should be within local or regional guidelines in adults during infusion. If the level is high, ensure the blood sample was drawn from an appropriate location. If the level exceeds for example 400 mg/dL (4.5 mmol/L) investigate secondary causes. Serum triglycerides should be assessed prior to the beginning of infusion in all patients.	Agree: 17 (100%)^b^ Do not agree: 0 Do not wish to answer: 0
13. When mechanical circulatory support systems (e.g. ECMO) or the use of cardiopulmonary bypass are required, the function of the membrane oxygenator should be closely overseen during lipid emulsion administration to monitor for the potential risk of clotting, which has occurred in very few cases. Additionally, when calculating the lipid intake, non-nutritional lipid sources (e.g. propofol) should be taken into account, and closely monitor triglyceride levels. Lipid emulsions may be administered as a continuous infusion over 12–24 h through a remote central venous line (prevent bolus application and infusion directly into the ECMO circuit).	Agree: 15 (83.3%) Do not agree: 0 Do not wish to answer: 3 (16.7%)
Surgical and hospitalized patients	
14. In adult surgical and hospitalized patients requiring parenteral nutrition, ILEs are an integral part of parenteral nutrition.	Agree: 18 (100%) Do not agree: 0 Do not wish to answer: 0
15. There is accumulating scientific evidence from clinical trials, systematic reviews, and meta-analyses to demonstrate that ILEs containing fish oil have clinically meaningful advantages over ILEs without fish oil when used in adult patients requiring parenteral nutrition including surgical and hospitalized patients with a favorable risk–benefit ratio. Furthermore, the use of ILEs containing fish oil as part of parenteral nutrition have shown to be cost-effective in these populations.	Agree: 16 (88.9%) Do not agree: 1 (5.6%) Do not wish to answer: 1 (5.6%)
16. In adult patients, the ILE dose is in general up to 1.5 g/kg/day (including non-nutritional lipid sources such as propofol). A minimum dose of ILEs should be given to prevent EFA deficiency.	Agree: 17 (94.4%) Do not agree: 1 (5.6%) Do not wish to answer: 0
17. Based on currently available clinical data, we recommend 0.1–0.2 g fish oil/kg/day, provided by lipid emulsions containing fish oil, for adult surgical and hospitalized patients requiring parenteral nutrition.	Agree: 16 (88.9%) Do not agree: 1 (5.6%) Do not wish to answer: 1 (5.6%)
18. The use of mixed lipid emulsions containing soybean oil, olive oil, MCTs and/or fish oil at the recommended dose has not been shown to lead to EFAD in clinical practice. A 100% fish oil ILE has also not been shown to lead to EFAD in clinical practice.	Agree: 17 (100%)^b^ Do not agree: 0 Do not wish to answer: 0
19. Based on currently available clinical data, there is no need to withhold or limit (for safety concerns) the use of ILEs containing fish oil for parenteral nutrition during the first week of parenteral nutrition.	Agree: 18 (100%) Do not agree: 0 Do not wish to answer: 0
20. Based on clinical studies, systematic reviews and meta-analyses, there is no evidence that ILEs containing fish oil increase the risk of coagulopathy or bleeding abnormalities.	Agree: 18 (100%) Do not agree: 0 Do not wish to answer: 0
21. The concentrations of triglycerides in serum should be within local or regional guidelines in adults during infusion. If the level is high, ensure the blood sample was drawn from an appropriate location. If the level exceeds for example 400 mg/dL (4.5 mmol/L) investigate secondary causes. Serum triglycerides should be assessed prior to the beginning of infusion in all patients.	Agree: 18 (100%) Do not agree: 0 Do not wish to answer: 0
22. In patients at low metabolic risk, consider initiation of parenteral nutrition if it is anticipated that the patient will be unable to attain 50–60% of goal energy and proteins after the first 5–7 days.	Agree: 15 (83.3%) Do not agree: 2 (11.1%) Do not wish to answer: 1 (5.6%)
23. In malnourished/high metabolic risk patients, consider early initiation of supplemental parenteral nutrition if enteral or oral nutrition is contraindicated or insufficient.	Agree: 16 (88.9%) Do not agree: 2 (11.1%) Do not wish to answer: 0
24. Although enteral nutrition is considered as the first line of treatment in severe pancreatitis, if the patient requires parenteral nutrition, ILEs are an integral part of this parenteral nutrition.	Agree: 18 (100%) Do not agree: 0 Do not wish to answer: 0
Supplemental parenteral nutrition	
32. Supplemental parenteral nutrition is a combination of oral/enteral nutrition and parenteral nutrition. It may be considered as a strategy with the intent to increase macronutrient delivery and to maintain/improve the nutritional status of patients such as critically ill (acute phase), surgical, and cancer patients if oral or enteral nutrition is insufficient. ILEs are an integral part of supplemental parenteral nutrition.	Agree: 17 (100%)^b^ Do not agree: 0 Do not wish to answer: 0
33. Administration of supplemental parenteral nutrition through a peripheral line can be considered over a short period of time when central line access is unavailable or as a bridge until central line access is available. ILEs are an integral part of peripheral parenteral nutrition.	Agree: 17 (94.4%) Do not agree: 1 (5.6%) Do not wish to answer: 0
ILEs in parenteral nutrition – practical handling aspects	
37. If using all-in-one admixtures, the maximum infusion duration is 24 h.	Agree: 18 (100%) Do not agree: 0 Do not wish to answer: 0
38. When ILEs are given independent of dextrose and amino acids, infusion duration of ILEs should not exceed 12 h to minimize the risk of contamination.	Agree: 18 (100%) Do not agree: 0 Do not wish to answer: 0
Request to organizations issuing guidelines and recommendations in parenteral nutrition	
39. Nutrition societies should issue guidelines and recommendations addressing clinical validity when performing a systematic review. Differences in inclusion/exclusion criteria and methodology can result in significant differences in outcomes and conclusions. Translation of systematic review conclusions into clinical guidelines is also affected by many factors including the intent of the convening body, geographical regulations impacting clinical options, and balance between clinical requirement and need for additional definitive evidence.	Agree: 18 (100%) Do not agree: 0 Do not wish to answer: 0

a These consensus statements were formulated and voted on by the expert panel (listed below) participating in the International Lipids in PN Summit 2022, and thus represent the collective opinion of the summit experts informed by scientific evidence. The consensus summit experts who attended this meeting were: Magnus Bäck, Sweden; Philip Calder, UK; Sarah Cogle, USA; Valerio Chiurchiù, Italy; David Evans, USA; Leah Gramlich, Canada; Martin Hersberger, Switzerland; Stanislaw Klek, Poland; Robert Martindale, USA; Stephen McClave, USA; Bettina Mittendorfer, USA; Manpreet Mundi, USA; Maurizio Muscaritoli, Italy; Reid Nishikawa, USA; Jayshil Patel, USA; Lorenzo Pradelli, Italy; Martin Rosenthal, USA; Charles Serhan, USA; Christian Stoppe, Germany; Kelly Tappenden, USA; Dan Waitzberg, Brazil; Malissa Warren, USA; Paul Wischmeyer, USA (note not all experts were present for all sessions of the meeting/voted on all of the consensus statements).

b One delegate skipped these questions.

ECMO, Extracorporeal membrane oxygenation; EFA, essential fatty acid; EFAD, essential fatty acid deficiency; ICU, intensive care unit; ILE, intravenous lipid emulsion; MCT, medium chain triglyceride.

### Parenteral nutrition: lipid choice

PN is considered to be a clinical management ‘cornerstone’ for patients with intestinal failure whose nutritional needs cannot be met via oral or enteral nutrient routes, particularly those undergoing major surgical procedures who are malnourished and/or with complex comorbidities (2). In these cases, nutritional support is particularly vital, from the prehabilitation phase for surgical patients through to the post-operative period, helping to alleviate the metabolic stress and inflammatory responses typically triggered by surgery. Several changes within the field of PN have emerged that can potentially benefit clinical outcomes for surgical patients, including a closer attention to glycemic control and a broader availability of ILEs, particularly mixes of lipids containing soybean oil, olive oil, medium-chain triglycerides (MCT), and fish oil (13). Fish oil contains long-chain omega-3 PUFAs (eicosapentaenoic acid [EPA] and docosahexaenoic acid [DHA]) (8). Omega-3 PUFA use within PN has recently gained considerable attention owing to multiple potential clinical benefits, including their anti-inflammatory effects, and ability to preserve muscle mass (4). They also act as precursors to specialized pro-resolving mediators (SPMs) – which play a key role in the active resolution of inflammation whilst supporting host immune-defense mechanisms (4). Taken together, these properties make omega-3 PUFAs an appealing choice as a PN component, particularly for surgeons and other healthcare practitioners looking to enhance postoperative recovery and decrease complication rates (13, 14).

In recent years, the knowledge base has evolved concerning if, how, and when to use ILEs as part of PN for surgical patients, most recently concerning lipid choice. Some early studies suggested harmful effects in trauma patients regarding the use of omega-6 ILEs in PN (*vs* no lipids), which worsened outcomes (e.g., infections) in trauma patients (15). However, these initial findings have since been questioned, as subsequent studies showed similar infection rates in patients receiving PN with or without ILEs (16, 17). Presently, best practice does not support that the use of ILEs in PN is a risk factor for sepsis in patients, as prior concerns were largely based on outdated and inconsistent literature (18). Thus, ILEs are generally considered to be an essential component of PN, providing crucial non-protein energy and essential fatty acids (8, 10, 19).

Ample evidence has emerged in recent years concerning better clinical outcomes for hospitalized/surgical patients when giving PN incorporating modern composite ILEs containing fish oil compared with older generation pure soybean-oil ILEs (6, 7, 20). For example, PN including ILEs containing fish oil (*vs* PN containing ILEs without fish oil) significantly reduced the risk of infection and sepsis by 40 and 56%, respectively, and length of both ICU and hospital stay by about 2 days (6) Furthermore, cost–benefit analyses have revealed that ILEs containing fish oil are a more cost-effective intervention than ILEs without fish oil (20). As such, the consensus statements (Table 1, statements 6–8) acknowledge that ILEs containing fish oil have clinically meaningful advantages over ILEs without fish oil in adult surgical and critically ill patients requiring PN, and are also a cost-effective intervention in these populations, solidifying their value in PN protocols across surgical settings.

### Role of lipids in critical illness: example of surgery-related intestinal failure

As part of the meeting, delegates examined typical instances where the choice of ILE might be particularly important. One such example was a case study of a patient with surgery-related intestinal failure (see Box 1). A retrospective review of the case identified two key learning points. These were, an undesirable lengthy delay in providing clinical nutrition (PN was started on day 8 after surgery), as well as a potentially detrimental delay in using an ILE as part of the initial PN prescription.

BOX 1 **Case study of surgery-related intestinal failure**

A 63-year-old male with a diagnosis of diverticulitis complicated by a chronic pelvis abscess was admitted for elective surgery (robotic sigmoidectomy with primary anastomosis, protecting loop ileostomy). He had a history of methamphetamine use and smoking, heart failure, COPD, pre-diabetes, coccidioidomycosis, severe pneumonia, and left upper lobectomy, as well as a poor diet (microwave meals and fast food) and low BMI (17.4).

On day 3 after surgery gas and stool production occurred via ileostomy, and the patient advanced to regular (oral) diet, but by day 5 was transferred to ICU for septic shock, CT scan showed residual free fluid/air, dilated loops of bowel, vasopressors given, elevated lactate levels (11 mmol/L). On day 6, an exploratory laparotomy had negative findings, the patient developed high nasogastic and ileostomy outputs (>3 L and > 6 L, respectively), and *Clostridium difficile* was detected in the ileostomy output. Vasopressors were weaned, and the patient remained mechanically ventilated and sedated with propofol. On day 8 after first surgery, PN was started. Initially, ILE was withheld while still receiving propofol; thereafter (by day 10), 250 mL SMOFlipid was added to the PN prescription to provide approximately 20 to 25% of estimated calorie needs (0.9 g/kg ILE and 0.13 g/kg fish oil/day). Monitoring for elevated liver function tests, hyperlipidemia, thrombocytopenia, and altered platelet aggregation was performed, as well as for essential fatty-acid deficiency (individual fatty acid levels and triene:tetraene ratio).

Once the location of the leak was identified from the large bowel and was considered contained, low-rate (20 mL/h) enteral nutrition was started. Full-dose PN prescription (including ILE) continued until enteral nutrition was titrated towards goal rate on postoperative day 15. The contained leak or gastrointestinal fistula remained low output. As the patient recovered from critical illness, oral nutrition was started with ongoing supplemental enteral nutrition. The patient was discharged on postoperative day 22 for a 2-week rehabilitation stay before returning home.

BMI, body-mass index; COPD, chronic obstructive pulmonary disease; CT, computed tomography; ILE, intravenous lipid emulsion; PN, parenteral nutrition.

Delays in providing clinical nutrition within the postoperative setting could be attributed to uncertainty regarding the optimal timing for starting PN after surgery when EN is not feasible, particularly when a patient is not malnourished at the time of surgery, as in this case. Unfortunately, early enteral nutrition was delayed by a complicated postoperative course, characterized by critical illness with sepsis, hemodynamic instability, high nasogastric output, and lack of distal enteral feeding access. In addition, by day 7 after surgery the patient had developed leakage of gastrointestinal contents from the abdominal surgical incision. When PN was started, an ILE was not added to the initial PN prescription owing to the patient receiving a small dose of non-nutritive lipids in the form of propofol (equivalent to approximately 200 lipids calories/day). Propofol was weaned on postoperative day 10, and ILE was then added to the PN prescription.

Regarding the learning point of the lengthy delay in starting clinical nutrition, it may have been beneficial to start PN as soon as the patient was hemodynamically stable because the prolonged course of critical illness and difficulty feeding enterally could have been predicted. When considering the second key learning point, in retrospect, it may have been beneficial to start giving at least a small dose of ILE whilst the patient was still being given propofol (given that ILEs should be considered an integral part of PN; see Table 1, statement 14), and that this ILE could ideally contain fish oil as a component (because of the potential clinical benefits associated with ILEs containing fish oil; see Table 1, statement 15).

The traditional viewpoint concerning ILE use in PN is that they are a major source of non-protein energy, lowering the amount of carbohydrate that needs to be provided as part of nutrition support (often allowing for better glycemic control), as well as providing essential fatty acids (thus preventing essential fatty-acid deficiency), and allowing the delivery of fat-soluble vitamins (8, 9, 21, 22). However, it is becoming increasingly evident that the different fatty-acid compositions of various ILEs can result in a range of biological effects, which may translate into changes in clinical outcomes (8). Thus, there have been some concerns about using ILEs (such as pure soybean-oil ILEs) containing a high omega-6 content, as mentioned previously, leading to guidelines recommending withholding or limiting the use of pure soybean-oil ILEs in the first week of PN in critically ill patients (11). However, the view of the summit attendees, based on currently available clinical data, was that there is not a need to withhold or limit (for safety concerns) the use of ILEs containing fish oil during the first week of PN (Table 1, statement 19). Furthermore, it was agreed that in adult patients, the ILE dose should, in general, be a maximum of 1.5 g/kg/day (including non-nutritional lipid sources such as propofol), and a minimum ILE dose should be given to prevent essential fatty acid deficiency (EFAD) (Table 1, statement 16). In particular, it was acknowledged that the use of mixed lipid emulsions containing soybean oil, olive oil, MCTs and/or fish oil have not been shown to lead to EFAD in clinical practice (Table 1, statement 18).

### Role of lipids in surgery: example of a trauma patient

In Box 2, a case study from the summit shows an example of a trauma patient in Ohio, USA, and their management, including nutritional support. The Ohio region is notable in that it has implemented a system of virtual nutrition support teams to cover multiple acute care hospital sites, helping to optimize PN and improving blood glucose control (23). This case study underscores several key learning points, such as the need for ongoing education regarding the importance of volume-based feeding, addressing enteral intolerance, and the important role played by supplemental PN in some cases, to achieve adequate caloric and protein intake. It is known that critically ill patients who received at least two-thirds of their prescribed caloric intake are much less likely to die than those receiving less than one-third of their prescription (odds ratio 0.67; 95% confidence interval 0.56–0.79; *p* < 0.0001) (24). Yet, as shown in this case study (Box 2), it is often difficult to meet patients’ energy requirements, even in centers with robust nutritional therapy teams in place, leading to cumulative energy deficits that can impair recovery and increase the likelihood of complications. One solution is supplemental PN (SPN) that can help to bridge the caloric gap. SPN has shown clinical benefits, such as lower nosocomial infection and ICU mortality rates (25, 26), resulting in this strategy being included in American and European guidelines (11, 12, 19, 27). Nevertheless, the clinical application of SPN has been heterogeneous internationally (25). Barriers to uptake of SPN include a lack of strong evidence regarding the optimal timing/specific criteria for starting SPN, an absence of SPN protocols, unfounded concerns about infection risks, inability to accurately assess energy requirements, and lack of understanding and education regarding the importance of cumulative energy deficits (25). The need for clearer and more practical guidance concerning SPN was also identified at the summit, with some experts recalling that many physicians have found aspects of nutritional society guidelines somewhat impractical, such as how and when to use SPN. Another issue that was highlighted at the summit was PN component shortages, which at times could lead to a tendency towards the provision of less than adequate nutritional dosing, resulting in potential nutrient deficiencies and impaired patient health.

BOX 2 **Case study of a trauma patient (motor vehicle collision)**

A 31-year-old male extricated from a car following a high-speed motor vehicle collision, with bilateral open femur fractures and in hemorrhagic shock (heart rate in the 120 s; blood pressure 88/56 mmHg), was taken the operating room initially for splenectomy, pelvic packing and external fixation of the pelvis.

The day after admission, a CT scan revealed an intraparenchymal haemorrhage and the patient was diagnosed with elevated ICP. At this stage the patient’s systolic pressure was in the 80s, and he was still receiving blood products on and off. The surgery team considered using norepinephrine if shock persisted, but hemoglobin levels stabilized (core temperature 35°C, hemoglobin 6.2 g/dL, lactate 6.8 mmol/L).

Immune-modulating EN delivering 1.5 kcal/mL containing arginine and omega-3 fatty acids was administered (Impact 1.5, Nestle) with a goal prescription of 1,560 mL (2,340 kcal)/day. However, actual average daily EN given was 858 mL (1,287 kcal)/day over the first 10 days. By that point (day 10 after admission) the patient developed ileus and pancreatic fistula (from the tail of the pancreas), and REE was measured at 3200 kcal/day, such that by this time the patient had a 10,530 kcal deficit (from prescribed goal) and a 19,000 kcal deficit according to the REE. At this stage, PN was ordered that included SMOFlipid (1.1 g/kg/day). The patient was supported for 10 days with SPN that reliably delivered 2040 kcal/day and continued on EN, as tolerated, with the recognition that the combination of SPN + EN as currently delivered would achieve the 3,200 kcal REE target. The patient was transferred out of the ICU and the pancreatic fistula resolved. By day 20 the patient was reliably tolerating full EN and SPN was discontinued in anticipation of discharge to rehabilitation.

This case highlighted several opportunities for improvement in our program that we are currently addressing. While we have a volume-based feeding protocol in place, the implementation of that protocol is not routine, and we need to better identify which patients should be started on volume-based protocols (or make a complete programmatic switch to volume-based feeds). With staff turnover we also need brief continuous education to emphasize the importance of volume-based feeding. There are also numerous opportunities to improve our protocol regarding the strategies to address enteral intolerance including small bore jejunal feeds and streamlined use of promotility agents.

CT, computed tomography; EN, enteral nutrition; ICP, intracranial pressure; PN, parenteral nutrition; REE, resting energy expenditure; SPN, supplemental parenteral nutrition.

### Chronic critical illness/persistent inflammation, immunosuppression and catabolism syndrome

In an environment of acute stress (e.g., during sepsis), multiple metabolic responses can arise, resulting in conditions such as systemic inflammatory response syndrome (SIRS), compensatory anti-inflammatory response syndrome (CARS), or PICS (persistent inflammation, immunosuppression and catabolism syndrome) (13). After an inflammatory insult such as sepsis, three main clinical courses may occur. Firstly, patients may recover fully. Secondly, SIRS and CARS can occur simultaneously, forming an overwhelming SIRS response, multiple organ failure, and fulminant death (13). Lastly, and most relevant to this topic, patients can enter a state of lingering chronic critical illness (CCI), with induced frailty, long-term disabilities, and indolent death (28). A sub-set of CCI patients with continued, smoldering, persistent state of inflammation, catabolism, and immunosuppression (i.e., PICS) is typical of the pathobiology in septic surgical ICU patients (28).

The epidemiology of chronic illness after severe trauma has been explored in a prospective observational study (*n* = 135 trauma ICU patients with hemorrhagic shock who survived beyond 48 h after injury) (29). Early mortality was low after severe trauma (79% exhibited a rapid recovery), but CCI was a common clinical course among survivors, as nearly one-fifth (19%) of patients surviving more than 48 h after severe trauma developed CCI. Patients with CCI tended to have poorer outcomes, such as high rates of in-hospital infectious and non-infectious complications, greater organ failure severity, and were associated with prolonged resource utilization. In addition, 56% of those developing CCI either died before discharge or had a ‘poor’ discharge disposition (discharge to a skilled nursing facility or long-term acute care facility), known to be associated with poor long-term outcomes (29).

The role of adequate nutritional support in the prevention of CCI-PICS was explored by a retrospective *post hoc* analysis of a sepsis database (28). This identified 56 CCI patients who received early, adequate nutritional support according to an established surgical ICU protocol (early EN, followed by PN or SPN if either were needed), and compared these cases with 112 matched patients who had a rapid recovery (28). Results showed that long-term outcomes in septic surgical ICU patients with CCI were not improved with early, adequate, evidence-based ICU nutrition, as many exhibited CCI-PICS. As such, the authors concluded that it was not feasible to ‘feed patients out of CCI-PICS’ and instead recommended further work into ‘novel adjuncts’ (such as omega-3 PUFAs and their SPM derivatives) to improve long-term outcomes in CCI-PICS populations (28). Currently, there is a lack of studies investigating potential effects of different ILE formulations in patients with CCI-PICS, though a scientific rationale may exist for these types of clinical trials.

Clearly, CCI-PICS is currently a common and highly morbid clinical trajectory, and is generally associated with fairly dismal outcomes. One potential solution to help prevent CCI-PICS and manage this response to injury is early identification that may allow targeted interventions to change the trajectory of this morbid phenotype (29). Furthermore, as we know that a persistent inflammatory response drives catabolism, it seems reasonable to use PN containing fish oil, when PN is indicated, as this may help to resolve inflammation and thus decrease the likelihood of CCI-PICS (13, 28). This approach is reflected in two consensus statements from the previous lipid summit, that stated “In high-risk, critically ill, adult patients (e.g., sepsis, ARDS, PICS), we recommend using fish-oil containing ILEs as part of the PN… in high-risk, critically ill, adult patients (e.g., sepsis, ARDS, and PICS), we recommend including fish-oil containing ILEs as part of PN in the first week of PN” (13). However, there was no specific mention of CCI-PICS in the consensus statements from the current lipid summit relevant to this topic. Nevertheless, these patients should be considered as part of the “Medical and surgical ICU patients” category, such that statements 7 and 8 apply, recommending the use of ILEs containing fish oil during the first week of ICU admission based on biological plausibility and associated clinical benefits, and clinically meaningful advantages over ILEs without fish oil (Table 1).

## Conclusion

The adverse physiological changes that occur following surgery and trauma can be partially mitigated by timely nutritional support, with PN being recommended when enteral or oral nutrition are not possible or insufficient. In recent years, the knowledge base has evolved concerning ILEs use as part of PN for surgical patients. In particular, the choice of ILE is now known to be a critical factor that can influence clinical outcomes in some patients. As such, for hospitalized/surgical patients, there is evidence for better clinical outcomes when giving PN incorporating modern composite ILEs containing fish oil compared with older generation pure soybean-oil ILEs. These benefits can include reduced risk of infection and sepsis, shorter duration of ICU and hospital stays, and superior cost effectiveness. Therefore, consensus statements from the international Lipids in PN Summit acknowledge that ILEs containing fish oil have clinically meaningful advantages over ILEs without fish oil in adult surgical and critically ill patients requiring PN, and are also a cost-effective intervention (Table 1, statements 6–8). Finally, the use of ILEs containing fish oil may provide particular benefits in special patient populations, including major trauma patients and those with CCI-PICS, though further research is needed to confirm this.
